# The Impact of Generative Artificial Intelligence Use on Perceived English Learning Achievement: The Roles of Use Behavior and Task–Technology Fit

**DOI:** 10.3390/bs16050643

**Published:** 2026-04-25

**Authors:** Zhongrui Wang, Shibao Guo

**Affiliations:** Werklund School of Education, University of Calgary, Calgary, AB T2N 1N4, Canada; zhongrui.wang1@ucalgary.ca

**Keywords:** generative artificial intelligence, use behavior, artificial intelligence self-efficacy, Task–Technology Fit

## Abstract

The rapid advancement of generative artificial intelligence (GAI) has intensified interest in its potential to support English learning in higher education. However, the mechanisms through which students’ perceptions and motivations translate into learning achievement remain unclear. Drawing on the Unified Theory of Acceptance and Use of Technology (UTAUT) and Task–Technology Fit (TTF) theory, this study investigates how undergraduate students’ use of GAI relates to perceived English learning achievement and under what conditions these associations are amplified. Using covariance-based structural equation modeling (CB-SEM), data from 537 undergraduate students across five public universities in China were analyzed. The findings indicate that performance expectancy, effort expectancy, facilitating conditions, perceived competitiveness, and artificial intelligence self-efficacy significantly predict GAI use. In turn, use behavior mediates their relationships with perceived English learning achievement. Task–Technology Fit further moderates the link between use behavior and learning achievement, with stronger associations observed when GAI functionalities are perceived as closely aligned with task requirements. These results highlight the importance of use behavior and task alignment in explaining how GAI is associated with students’ perceived English learning achievement and extend technology acceptance research within AI-supported language learning contexts.

## 1. Introduction

The rapid development of generative artificial intelligence (GAI) has reshaped technology-enhanced language learning in higher education (K. Qu & Wu, 2024). Unlike earlier computer-assisted language learning (CALL), which relied on predefined materials and limited interaction modes, GAI systems can generate context-relevant language output, provide adaptive feedback, and guide learners in sustained dialog (Gruba, 2004; Tan et al., 2025; Yeh, 2024). These capabilities support a broad range of language-learning activities, including writing, revision, vocabulary expansion, and language clarification (Haleem et al., 2022; Johnston et al., 2024; Lee et al., 2023). GAI is increasingly seen as an interactive learning partner that extends practice beyond the classroom (B. Liu, 2023; Waluyo & Kusumastuti, 2024).

Yet recognizing these advantages does not, by itself, translate into learning outcomes (Castañeda & Selwyn, 2018; Holmes & Miao, 2023; Selwyn, 2016). Much of the existing research adopts an outcome-oriented perspective and seldom asks how or why those outcomes arise (Waluyo & Kusumastuti, 2024). Traditional technology acceptance factors such as perceived usefulness and perceived ease of use dominate the literature, while motivational pressures and AI-specific capability perceptions receive comparatively little attention (Acosta-Enriquez et al., 2024; Moghavvemi & Jam, 2025; Xue et al., 2024). Some studies further assume that more GAI use will automatically produce better results, overlooking the task-dependency inherent in both language learning and GAI technology (Alamri et al., 2020; Al-Dokhny et al., 2024). This study addresses these gaps by integrating the Unified Theory of Acceptance and Use of Technology (UTAUT) with Task–Technology Fit (TTF) theory, positioning use behavior as a mediating mechanism and Task–Technology Fit as a moderating condition (Al-Rahmi et al., 2022; Jebrila et al., 2024).

Language learning is a complex and dynamic process influenced by many factors, including cognition, society, and context (Esfandiari & Arefian, 2025; Marek & Wu, 2014; Ushioda, 2015). At the undergraduate level, English learning usually requires students to develop vocabulary, grammar, reading, writing, listening, and speaking skills while coping with increasingly interdisciplinary academic demands (Nation, 2001; Yeh, 2024). Computer-assisted language learning (CALL) has long been adopted in higher education to address these needs (Gruba, 2004; Yeh, 2024). GAI represents a qualitative shift. Unlike traditional CALL applications, GAI systems can generate context-relevant output and engage learners in sustained, comprehensible interactive dialog (Yeh, 2024).

For undergraduates, GAI can serve as a learning assistant that provides explanations, examples, grammar correction, idea generation, and practice with task-specific language (Haleem et al., 2022; Johnston et al., 2024). Through immediate feedback and adaptive responses, GAI can help learners identify weaknesses and refine their language use over successive attempts, thereby enhancing both accuracy and fluency (Lee et al., 2023). Learners often perceive AI-generated suggestions as relevant, easy to understand, and helpful, especially in academic writing and revision tasks (Dai et al., 2023). Beyond skill development, GAI is also positively associated with students’ learning engagement, confidence, and willingness to participate (J. Liu, 2024; H. Liu & Fan, 2024). The conversational and responsive nature of these tools has been shown to reduce language learning anxiety by providing a low-pressure environment for trial and error (Saptiany et al., 2025), a feature particularly important in higher education contexts where students may feel anxious about academic performance and peer comparisons (Bibauw & Dizon, 2025). Students who incorporate GAI into their study routines have demonstrated gains in vocabulary acquisition, grammatical accuracy, and writing quality compared with those relying solely on traditional materials (Rojas et al., 2026).

However, these benefits come with notable concerns. Questions of academic integrity arise when students submit AI-generated content without genuine cognitive engagement (Bittle & El-Gayar, 2025; Kofinas et al., 2025). Heavy reliance on GAI may also weaken the development of critical thinking and independent problem-solving skills, as learners risk becoming passive recipients of AI-generated answers rather than active constructors of knowledge (Premkumar et al., 2024). The accuracy and reliability of AI-generated language feedback remain inconsistent, which may reinforce errors or provide misleading guidance in certain learning contexts (Mortlock & Lucas, 2024). These challenges underscore the need to move beyond general claims about GAI’s benefits and to investigate the specific conditions under which its use is associated with meaningful learning outcomes.

Although existing evidence generally indicates positive associations between GAI and learning outcomes, current studies often focus on outcome comparisons or learners’ perceptions (Waluyo & Kusumastuti, 2024; H. Liu et al., 2025; Y. Wang et al., 2025). Less attention has been paid to the underlying mechanisms through which students’ interactions with GAI are shaped by their beliefs, motivation, and learning environment, nor to the conditions that may constrain these benefits. Such gaps call for a deeper investigation into the factors associated with students’ adoption and use of GAI.

Despite the growing body of research, several key gaps remain. Few studies have examined theoretically how learners’ perceptions and beliefs translate into academic outcomes (Sun & Zhou, 2024; Waluyo & Kusumastuti, 2024; Y. Wang et al., 2025), and use behavior is often treated as a peripheral outcome rather than a core mediating mechanism linking acceptance-related factors to achievement (Xue et al., 2024). Motivational pressures and capability differences that may shape the strategic and sustained application of GAI also remain largely unexplored (Moghavvemi & Jam, 2025; Xue et al., 2024). At the same time, most work implicitly equates greater use with better outcomes, without considering whether the tool fits the task at hand (Alamri et al., 2020; Al-Dokhny et al., 2024).

Based on the above research status, this study intends to address the following questions:

(1) Are students’ cognitions of generative artificial intelligence, support conditions, and learning environment factors related to their use of GAI tools for English learning?
(2) Is students’ use of generative artificial intelligence in English learning linked to their perceived English learning achievement?
(3) When generative artificial intelligence is highly matched with learning tasks, is the relationship between use behavior and perceived English learning achievement stronger?

## 2. Research Hypotheses

### 2.1. Performance Expectancy (PE)

Performance expectancy refers to the degree to which an individual believes that a technology can improve task performance (Duong et al., 2024; Venkatesh et al., 2003; Rahi et al., 2019). In educational contexts, performance expectancy reflects learners’ judgments about whether technological tools improve learning efficiency and academic outcomes (Scherer et al., 2019; Strzelecki, 2023b). Existing studies indicate that performance expectancy is an important predictor of technology use, especially in AI-supported learning environments (Dwivedi et al., 2019; Duong et al., 2024).

### 2.2. Effort Expectancy (EE)

Effort expectancy reflects individuals’ perceptions of the ease of use and operational complexity of a technology (Acosta-Enriquez et al., 2024; Venkatesh et al., 2003). Higher perceived ease of use can reduce barriers to technology adoption and promote continued use (Oyewole, 2018; Song & Song, 2023). Given that GAI features natural language interaction and an intuitive interface (S. Kim et al., 2023), perceived ease of use is expected to enhance students’ use of the technology.

### 2.3. Facilitating Conditions (FC)

Facilitating conditions refer to individuals’ perceptions of whether environmental and technical support help technology use (Sha et al., 2025; Venkatesh et al., 2003). Existing studies indicate that access to resources and institutional support are important external factors that drive technology use (Abbad, 2021; Mahande & Malago, 2019; Rumangkit et al., 2023).

### 2.4. Perceived Competitiveness (PC)

Perceived competitiveness reflects students’ subjective perceptions of academic competition and pressure from performance comparisons (Becker & Börnert-Ringleb, 2025). Higher perceived competitiveness typically motivates learners to seek strategic resources to improve performance (Bailey et al., 2024; Murayama & Elliot, 2012). In the GAI context, this motivational pressure may translate into a higher tendency to use the technology.

### 2.5. Artificial Intelligence Self-Efficacy (AISE)

Artificial intelligence self-efficacy refers to an individual’s belief in their ability to effectively use AI tools to complete learning tasks (Y. Wang & Chuang, 2024). Self-efficacy has been widely shown to be a key factor in technology adoption and continued use (Bandura, 1997; Obenza et al., 2024; Rodríguez-Ruiz et al., 2025).

### 2.6. Use Behavior (UB)

Use behavior refers to the extent to which learners use technology in a sustained and meaningful way in learning activities (Ashraf et al., 2025; Williams et al., 2015). In technology-enhanced learning research, use behavior is regarded as a key mechanism linking acceptance factors to learning outcomes (Venkatesh et al., 2003; Ashraf et al., 2025).

### 2.7. Perceived English Learning Achievement (ELA)

English learning achievement reflects learners’ development across key dimensions of English language proficiency (Dahri et al., 2024; Durán, 2008). Both theoretical and empirical research indicate that learning outcomes depend on sustained engagement and opportunities for practice (Alqarni, 2023; Boekaerts, 2016). In this study, English learning achievement was operationalized through students’ self-reported perceptions of their learning improvement, encompassing gains in overall English proficiency, comprehension, ease of learning, and learning efficiency.

### 2.8. Task–Technology Fit (TTF)

Task–Technology Fit refers to the degree of alignment between technological functions and task demands (Goodhue & Thompson, 1995; Q. Wang et al., 2026). TTF theory suggests that technology improves performance only when it effectively supports the task (Al-Rahmi et al., 2022).

Based on the above theoretical and empirical foundations, the following hypotheses are proposed:

**H1.**  *Performance expectancy is positively related to students’ use behavior of GAI.*

**H2.**  *Effort expectancy is positively related to students’ use behavior of GAI.*

**H3.**  *Facilitating conditions are positively associated with students’ use behavior of GAI.*

**H4.**  *Perceived competitiveness is positively linked to students’ use behavior of GAI.*

**H5.**  *Artificial intelligence self-efficacy is positively related to students’ use behavior of GAI.*

**H6a–e.**  *Use behavior mediates the relationships between the five antecedent variables (performance expectancy, effort expectancy, facilitating conditions, perceived competitiveness, and artificial intelligence self-efficacy) and perceived English learning achievement.*

**H7.**  *Students’ use behavior of generative artificial intelligence is positively associated with their perceived English learning achievement.*

**H8.**  *Task–Technology Fit positively moderates the relationship between use behavior and perceived English learning achievement; when Task–Technology Fit is higher, the positive relationship between use behavior and achievement will be stronger.*

**Figure 1:**
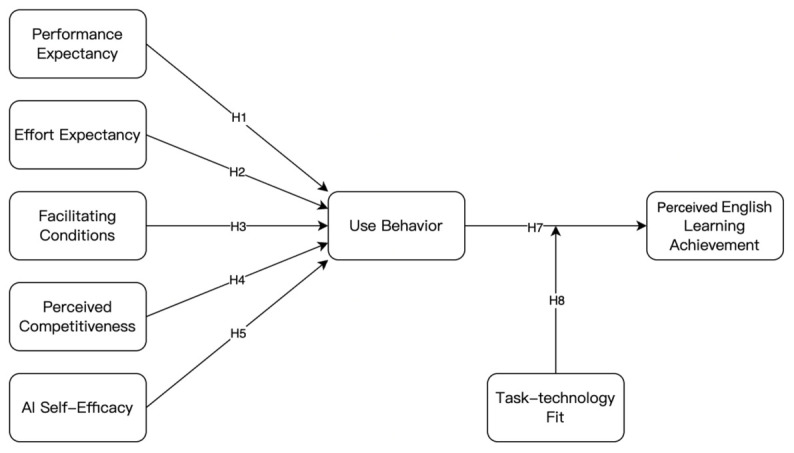
The finalized research model. Use behavior functions as a mediating mechanism between the antecedent variables and perceived English learning achievement (H6a–H6e). **Source:** Authors’ own elaboration.

The finalized research model integrating these hypotheses is presented in Figure 1.

## 3. Materials and Methods

### 3.1. Participants

The participants in this study came from five comprehensive public universities in China, including two Project 985 universities, two Project 211 universities, and one regular first-tier university. Project 985 and Project 211 refer to national initiatives aimed at strengthening a selected group of research-intensive universities in China, roughly comparable to elite or research-focused universities in other higher education systems (Costa & Zha, 2020; Lin & Wang, 2022). To ensure the validity and reliability of the data, this study did not provide monetary or material incentives and adopted strict inclusion criteria. Participants were required to be currently enrolled at the undergraduate level, have English as a second language, and have experience using GAI to support English learning. Before entering the questionnaire, all participants were required to read the study purpose, participation rights, and privacy protection measures, and confirm voluntary participation through an implied consent procedure (implied consent). Data were collected through the major Chinese online survey platform “Wenjuanxing” (Questionnaire Star; https://www.wjx.cn/). The study used an online convenience sampling method. The researcher posted an anonymous questionnaire link, recruitment poster, and study information in English-learning WeChat groups at the five universities to ensure that participants could fully understand the study content and response requirements. A total of 555 questionnaires were collected. Of the 555 returned questionnaires, 18 were identified as invalid and excluded based on two predetermined screening criteria: (a) completion time below 100 s, indicating insufficient engagement with the survey items (*n* = 10), and (b) selecting the same response option for more than 10 consecutive items, suggesting careless or patterned responding (*n* = 8). The final analytic sample comprised 537 valid questionnaires, yielding an effective response rate of 96.8%. Detailed demographic information is shown in Table 1.

### 3.2. Instruments

The questionnaire included two parts: demographic information (gender, grade, and field of study) and scales measuring eight core constructs in the research model. The second part contained a total of 32 items used to measure performance expectancy (PE), effort expectancy (EE), facilitating conditions (FC), perceived competitiveness (PC), AI self-efficacy (AISE), use behavior (UB), Task–Technology Fit (TTF), and perceived English learning achievement (ELA), with each construct consisting of 3–5 items. All variables were measured using a five-point Likert scale (1 = strongly disagree, 5 = strongly agree), and the original version of the questionnaire was in Chinese. The specific items and sources are shown in Table 2.

One item in the Use Behavior scale (UB3: “I intend to continue using GAI for English learning”) captures continuance commitment rather than current usage. Because all participants had prior GAI experience, UB3 reflects experience-based behavioral persistence rather than speculative pre-adoption intention (Lavidas et al., 2024; Sha et al., 2025). The reliability analysis further confirmed that removing UB3 would reduce the Cronbach’s alpha from 0.833 to 0.775, indicating that the item contributes positively to the construct’s internal consistency.

### 3.3. Ethical Considerations

This study strictly followed the Tri-Council Policy Statement: Ethical Conduct for Research Involving Humans (TCPS2) and the ethical requirements of the University of Calgary Research Ethics Board (REB25-1877). Before the formal start of the study, all procedures, instruments, and data collection methods had been approved through ethics review. Participation was entirely voluntary. Informed consent was obtained through implied consent, and participants could withdraw at any time before submitting the questionnaire. This study involved minimal risk and did not collect any personally identifiable information. All data were anonymized and securely stored in accordance with institutional data protection guidelines.

### 3.4. Data Analysis

Data analysis was conducted using SPSS Statistics 31 and AMOS 31. Prior to hypothesis testing, the reliability and convergent validity of the measurement scales were assessed using Cronbach’s alpha (α), composite reliability (CR), and average variance extracted (AVE). Harman’s single-factor test was employed as a preliminary check for common method bias (CMB), and discriminant validity was evaluated using the Fornell–Larcker criterion. Demographic group differences were examined using independent-samples *t*-tests and one-way ANOVA.

Hypothesis testing involved three complementary techniques. Covariance-based structural equation modeling (CB-SEM) was used to evaluate the overall measurement model through confirmatory factor analysis (CFA), establishing construct validity, reliability, and model fit, and to examine the structural relationships among the latent variables. CB-SEM is well-suited for theory-driven research involving multiple latent constructs and allows simultaneous estimation of measurement and structural parameters (Kline, 2023). Hierarchical regression was then employed to test the moderating effect of Task–Technology Fit on the relationship between use behavior and perceived English learning achievement. Interaction effects are more transparently examined in a regression framework, where the incremental variance explained by the interaction term can be directly assessed through changes in R^2^ (Aiken et al., 1991; Hayes, 2022). Finally, the PROCESS macro v5.0 for SPSS (Model 4) was used to estimate the indirect (mediation) effects of use behavior with bias-corrected bootstrapped confidence intervals (5000 samples) (Hayes, 2022). Bootstrapping is the recommended approach for testing mediation because it does not assume normality of the sampling distribution of the indirect effect (Hayes, 2022). This sequential strategy allows each method to address a specific analytical question while maintaining coherence across the overall model testing process. Recent research on technology adoption in AI-supported contexts has adopted similar combinations of CB-SEM with PROCESS-based moderation and mediation analyses (Jiao & Cao, 2024; Liang et al., 2023).

To ensure analytical traceability across the sequential analyses, the following procedural details are provided. After the measurement model was evaluated through CFA within CB-SEM, composite scores for each construct were computed as the arithmetic mean of the corresponding items (e.g., the composite score for performance expectancy was computed as the mean of PE1–PE4; all items were measured on the same 5-point Likert scale). These composite scores served as the observed inputs for the subsequent hierarchical regression and PROCESS macro analyses. For the moderation analysis, use behavior (UB) and Task–Technology Fit (TTF) were mean-centered prior to constructing the UB × TTF interaction term, in order to reduce non-essential multicollinearity between the interaction term and its constituent variables (Aiken et al., 1991; Hayes, 2022). Standardized regression coefficients (β) are reported to facilitate comparison of effect magnitudes across predictors. The transition from latent to observed variables across the analytical sequence is a deliberate methodological choice. CB-SEM provides a rigorous assessment of measurement quality and overall structural fit (J. Hair & Alamer, 2022; Kline, 2023). Regression-based moderation and PROCESS-based mediation analyses provide a more transparent presentation of incremental variance (ΔR^2^), simple-slope interpretation of interaction effects, and bootstrap-based inference for indirect effects, which together complement the latent-variable evidence obtained in CB-SEM (Aiken et al., 1991; Hayes, 2022).

Prior to hypothesis testing, the statistical assumptions underlying the chosen analyses were examined. For CB-SEM with ML estimation, univariate normality was assessed through the skewness and kurtosis of each composite variable; all values fell within acceptable ranges (|skewness| ≤ 0.235; |kurtosis| ≤ 0.415), below the thresholds of 2 and 7 (J. F. Hair et al., 2019; Maydeu-Olivares et al., 2024). For the hierarchical regression analyses including the UB × TTF interaction term (Model 6), all variance inflation factors ranged from 1.018 to 1.555, below the threshold of 5 (J. F. Hair et al., 2019; Staffini et al., 2022). The mean-centered UB × TTF term yielded a VIF of 1.033, confirming that mean-centering effectively reduced non-essential multicollinearity. Examination of standardized residual plots indicated approximately normal residuals, with no extreme values suggestive of influential outliers, and no evidence of heteroscedasticity.

## 4. Results

**Figure 2:**
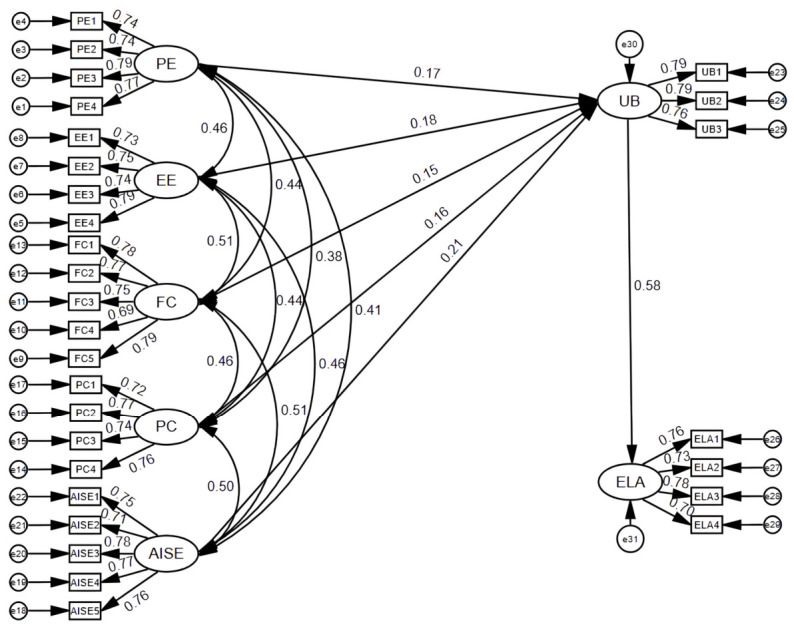
The measurement model. **Source:** Authors’ own elaboration.

This study adopted covariance-based structural equation modeling (CB-SEM) to evaluate the proposed research model and test the hypothesized relationships. CB-SEM is suitable for theory-driven research and aims to examine relationships among latent constructs and evaluate overall model fit (J. F. Hair et al., 2017; Kline, 2023). The specific measurement model is shown in Figure 2.

### 4.1. Common Method Bias Analysis

This study used Harman’s single-factor test to assess common method bias (CMB) and conducted exploratory factor analysis using SPSS 31. The results showed that the seven factors derived from the construct data explained 64.20% of the total variance, and the variance explained by a single factor ranged from 4.54% to 31.78%, which did not exceed the maximum threshold of 40%. Therefore, Harman’s single-factor test was passed (Fuller et al., 2016; J. Hair & Alamer, 2022). See Table 3 for details.

Harman’s single-factor test serves only as a preliminary screening tool and has known limitations in detecting common method bias (Podsakoff et al., 2003). As additional evidence, the hypothesized eight-factor CFA model demonstrated good fit, indicating that the eight constructs were empirically distinguishable under confirmatory analysis. The discrepancy between the seven-factor EFA solution and the eight-factor theoretical model is attributable to partial overlap between UB and ELA, whose items loaded onto the same component in the exploratory analysis. This overlap is theoretically understandable, as students who use GAI more frequently may also tend to report higher perceived learning improvement, creating shared variance between the two constructs. Under confirmatory analysis with theoretically informed constraints, UB and ELA were clearly differentiated.

Nevertheless, it should be acknowledged that both the EFA and CFA evidence reported here derive from the same single-source, single-wave self-report dataset. While the empirical distinguishability of constructs supported by CFA mitigates concerns about extreme common method variance, it does not eliminate the threat entirely. Some degree of common method variance therefore cannot be fully ruled out, particularly given the conceptual and empirical proximity between use behavior and perceived English learning achievement, whose items loaded onto the same component in the unrotated EFA solution.

### 4.2. Group Difference Analysis

This study examined differences in the core research variables across different demographic characteristics, specifically gender, grade, and field of study. The detailed results are shown in Table 4.

Regarding gender differences, the mean levels of male and female students were relatively close across most variables, and none of the differences reached a significant level (*p* > 0.05). A significant difference was observed only in use behavior (t = −2.010, *p* < 0.05), with female students scoring slightly higher than male students. Regarding grade differences, performance expectancy, effort expectancy, facilitating conditions, perceived competitiveness, AI self-efficacy, and use behavior all showed significant differences across grades (F values ranged from 2.667 to 8.214, *p* < 0.05), though effect sizes were small (η^2^ ranged from 0.015 to 0.044). Bonferroni post hoc comparisons indicated that the differences were primarily between first-year students and students in higher grades. Specifically, first-year students scored significantly lower than third-year students on performance expectancy (*p* = 0.013), lower than second-, third-, and fourth-year students on effort expectancy (all *p* < 0.01), facilitating conditions (all *p* < 0.01), and use behavior (all *p* < 0.01), and lower than third- and fourth-year students on AI self-efficacy (*p* = 0.008 and *p* = 0.001, respectively). No significant pairwise differences were found for perceived competitiveness. Task–Technology Fit and perceived English learning achievement did not show significant differences across grades (*p* > 0.05). Regarding field of study differences, a significant difference was found only for use behavior, with students from a social science background scoring significantly higher than students from a natural science background (t = −2.051, *p* < 0.05). These results suggest that while some group differences were statistically significant, their practical significance was limited, as effect sizes for gender and field of study comparisons (Cohen’s d) ranged from 0.018 to 0.177, and effect sizes for grade comparisons (η^2^) ranged from 0.009 to 0.044, all indicating small practical effects.

Based on the above results, gender, grade, and field of study showed differential patterns on some variables. Therefore, they were included as control variables in subsequent regression and structural model analyses to reduce the potential interference of demographic factors on the relationships among the core variables.

### 4.3. Descriptive Statistics and Reliability and Validity

This study evaluated the measurement model by examining construct reliability, convergent validity, and discriminant validity (J. Hair & Alamer, 2022). To assess construct reliability, Cronbach’s alpha and composite reliability (CR) were calculated. Cronbach’s alpha ranged from 0.794 to 0.869, all exceeding the recommended threshold of 0.70, indicating good internal consistency. Composite reliability ranged from 0.799 to 0.870, also exceeding the suggested cutoff value of 0.70 (J. F. Hair et al., 2017; J. Hair & Alamer, 2022). These results confirm that all constructs have adequate reliability.

Convergent validity was assessed by examining standardized factor loadings and the average variance extracted (AVE) (J. F. Hair et al., 2017; J. Hair & Alamer, 2022). All factor loadings exceeded the recommended value of 0.70, indicating strong associations between the observed indicators and their corresponding latent constructs (Barrett et al., 2021). AVE values ranged from 0.550 to 0.611, all exceeding the minimum threshold of 0.50 (Fornell & Larcker, 1981; J. Hair & Alamer, 2022). Given these results, the convergent validity of all constructs was well established. Detailed data are shown in Table 5.

This study used the Fornell–Larcker criterion to assess discriminant validity (Fornell & Larcker, 1981; J. Hair & Alamer, 2022). The square roots of the AVE values for each construct were greater than the corresponding inter-construct correlation coefficients, meeting the requirements of the Fornell–Larcker criterion (Fornell & Larcker, 1981; J. Hair & Alamer, 2022). These results indicate that each construct is empirically distinct from the other constructs. Detailed data are shown in Table 6.

### 4.4. Model Fit

This study employed covariance-based structural equation modeling (CB-SEM) to examine the hypothesized relationships among latent variables, with particular attention to the significance and magnitude of standardized path coefficients as well as the explanatory power of the model. Prior to conducting path analyses, the overall fit of the structural model was evaluated.

The results indicated a good model fit. Specifically, the chi-square to degrees of freedom ratio was acceptable (χ^2^/df = 1.736), falling below the recommended threshold of 3.0 (Kline, 2023). All incremental fit indices met or exceeded commonly accepted criteria, including GFI = 0.922, IFI = 0.964, TLI = 0.959, and CFI = 0.964 (L. T. Hu & Bentler, 1999). The root mean square error of approximation (RMSEA) was 0.037, indicating good model fit and parsimony (Browne & Cudeck, 1992). Detailed fit indices are reported in Table 7.

### 4.5. Main Effects and Moderation Effects

To test the main and moderating effects proposed in the research hypotheses, this study employed hierarchical regression and constructed six regression models. The analysis first used use behavior as the dependent variable to examine the predictive effects of technology acceptance-related variables and Task–Technology Fit. Subsequently, perceived English learning achievement was used as the dependent variable to further examine the mediating role of use behavior and the moderating effect of Task–Technology Fit. The detailed results are presented in Table 8.

Models 1 to 3 were used to examine the predictors of use behavior. In Model 1, only control variables were included. The results indicated that year of study was significantly positively associated with use behavior (β = 0.193, *p* < 0.001), whereas the effects of disciplinary background (β = 0.084, *p* < 0.05) and gender were relatively weaker (β = 0.096, *p* < 0.05). In Model 2, the inclusion of the core independent variables led to a substantial increase in explanatory power. The results showed that performance expectancy (β = 0.144, *p* < 0.001), effort expectancy (β = 0.161, *p* < 0.001), facilitating conditions (β = 0.121, *p* < 0.01), perceived competitiveness (β = 0.135, *p* < 0.001), and artificial intelligence self-efficacy (β = 0.170, *p* < 0.001) all had significant positive effects on use behavior. In Model 3, Task–Technology Fit was further included and was found to be positively associated with use behavior (β = 0.127, *p* < 0.01).

Models 4 and 5 used perceived English learning achievement as the dependent variable. In Model 4, only control variables were entered, and the results indicated that year of study was the only significant predictor of perceived English learning achievement (β = 0.091, *p* < 0.05). In Model 5, after adding the core independent variables and use behavior, the results showed that performance expectancy (β = 0.155, *p* < 0.001), facilitating conditions (β = 0.154, *p* < 0.001), perceived competitiveness (β = 0.104, *p* < 0.01), and artificial intelligence self-efficacy (β = 0.156, *p* < 0.001) were significantly positively associated with perceived English learning achievement, whereas the effect of effort expectancy was relatively weaker (β = 0.093, *p* < 0.05). At the same time, use behavior had a significant positive effect on perceived English learning achievement (β = 0.178, *p* < 0.001).

Model 6 further examined the moderating effect of Task–Technology Fit. After including Task–Technology Fit and its interaction term with use behavior, the results indicated that the interaction term was significantly positive (β = 0.114, *p* < 0.001), indicating that Task–Technology Fit significantly moderated the relationship between use behavior and perceived English learning achievement.

To further interpret the moderating effect of Task–Technology Fit, a simple slopes analysis was conducted, with TTF evaluated at the 16th, 50th, and 84th percentiles of the moderator distribution, corresponding to low, median, and high levels of perceived Task–Technology Fit, which is the default in the current version of PROCESS and a widely used alternative to the ±1 SD convention (Aiken et al., 1991; Hayes, 2022). The conditional effect of use behavior on perceived English learning achievement was positive and significant at all three levels of TTF, and increased monotonically as TTF increased: at low TTF (16th percentile), the simple slope was 0.19 (SE = 0.05, t = 3.72, *p* < 0.001, 95% CI [0.09, 0.29]); at median TTF (50th percentile), the simple slope was 0.30 (SE = 0.04, t = 7.75, *p* < 0.001, 95% CI [0.22, 0.37]); and at high TTF (84th percentile), the simple slope increased to 0.45 (SE = 0.06, t = 8.07, *p* < 0.001, 95% CI [0.34, 0.56]). This pattern indicates that the strength of the positive association between GAI use behavior and perceived English learning achievement becomes stronger as students’ perceived Task–Technology Fit becomes higher, providing further support for H8.

### 4.6. Mediation Effects

This study used mediation analysis to examine whether, when undergraduate students use GAI for English learning, use behavior functions as a psychological-behavioral mechanism linking the key antecedent variables to perceived English learning achievement. The analysis employed the SPSS PROCESS macro (Hayes, 2022) and applied a simple mediation model (Model 4) (Hayes, 2022). In this model, each antecedent variable served as the independent variable (X), use behavior served as the mediator (M), and perceived English learning achievement served as the dependent variable (Y). To evaluate the significance of the indirect effects, this study used a bootstrap method with bias-corrected 95% confidence intervals (CI). See Table 9 for details.

Across the five antecedent variables, the results consistently indicated that use behavior served as a significant mediating variable linking each antecedent to perceived English learning achievement. The confidence intervals for all five indirect paths did not include zero, indicating that the mediation effects remained robust under the bootstrapping tests. Importantly, the proportion of the mediation effects was nontrivial: the relative mediation effects ranged from 28.7% to 34.4%, suggesting that a substantial portion of each antecedent’s effect on perceived English learning achievement was transmitted through students’ use of GAI tools. The combined mediation results further supported this pattern. Each of the five hypothesized indirect effects was estimated through a separate PROCESS Model 4 analysis with the corresponding antecedent as the independent variable. Across all five models, the findings were consistent with partial rather than full mediation across the five hypothesized pathways (H6a–H6e).

Comparing the five mediation pathways, all five antecedent variables showed statistically significant mediation effects, further confirming that use behavior is a stable mechanism in the proposed model. The mediation proportions indicated that effort expectancy (34.4%) and perceived competitiveness (33.4%) had the strongest mediation effects, whereas facilitating conditions (28.7%) had a relatively weaker but still meaningful mediation effect. This implies that, in this sample, psychological accessibility (ease of use) and motivational pressure (competitiveness) may primarily translate into achievement by driving students’ engagement with GAI, whereas environmental support may function more as a necessary facilitating condition, still requiring other motivational factors to trigger high levels of engagement.

## 5. Discussion

### 5.1. The Limited Impact of Demographic Variables

Demographic variables showed limited but contextually meaningful differences in generative artificial intelligence-supported English learning. Background characteristics are not the primary factors associated with perceived English learning achievement, and their influence is mainly reflected in use behavior. Regarding the core constructs, gender differences were largely nonsignificant, and female students reported slightly higher levels of GAI use. This is consistent with previous findings that female learners demonstrate stronger strategic engagement in language learning contexts (Alhaysony, 2017; Ashraf et al., 2025; Mahmud & Sahril, 2018; Salahshour et al., 2013). Grade differences were mainly reflected in acceptance-related variables. Students in higher grades reported higher performance expectancy and stronger tendencies to use GAI; however, perceived English learning achievement and Task–Technology Fit did not differ significantly across grades. This suggests that accumulated experience may enhance confidence and acceptance, but it does not necessarily correspond to better learning outcomes. This finding is opposite to the results reported by Ashraf et al. (2025), who found that the core acceptance variables did not differ across grades. Field of study showed slight differences in use behavior, with social science students reporting slightly higher levels of use, but there was no systematic advantage in learning outcomes.

### 5.2. Factors Associated with Students’ Use of GAI

Students’ acceptance beliefs, motivational pressure, and perceived capability regarding generative artificial intelligence are closely related to their self-reported use behavior. The findings support the explanatory power of technology acceptance theory in GAI-supported language learning contexts. Performance expectancy, effort expectancy, facilitating conditions, and artificial intelligence self-efficacy significantly predicted students’ GAI use behavior, reinforcing the applicability of the UTAUT framework in AI-enhanced learning environments (Foroughi et al., 2023; Habibi et al., 2023; Strzelecki, 2023a, 2023b). Notably, artificial intelligence self-efficacy emerged as a particularly strong predictor, indicating that perceived capability is not merely a background characteristic but a key factor in sustained engagement. This is consistent with the findings of Ren et al. (2026) and Tailor and Tailor (2025). In addition, incorporating perceived competitiveness extended the traditional acceptance model by capturing motivational dynamics in high-pressure academic environments. Students who perceived greater academic competition were more likely to strategically adopt GAI as a resource to improve achievement, which echoes the findings of Yan et al. (2025) and Zhou et al. (2024) and highlights more pronounced patterns in AI-mediated language learning.

### 5.3. The Significant Mediating Role of Use Behavior

Use behavior, which reflects both current use and experience-based behavioral persistence, served as a significant mediator linking technology-acceptance-related factors to perceived English learning achievement. The mediation analysis indicated that use behavior significantly mediated the relationships between all five independent variables, performance expectancy, effort expectancy, facilitating conditions, perceived competitiveness, and artificial intelligence self-efficacy, on perceived English learning achievement. These findings support prior research by Ashraf et al. (2025), Song and Song (2023), Yu (2023), Zeb et al. (2025), and Huallpa (2023). This pattern indicates that positive cognitions alone are insufficient to be associated with perceived learning outcomes, as they may be realized through sustained and meaningful engagement with AI tools. The perceived benefits students derive from GAI appear to depend not simply on whether they value or trust the technology, but on whether these beliefs translate into sustained use, which provides opportunities for practice, feedback, and language development.

### 5.4. The Important Moderating Role of Task–Technology Fit

Task–Technology Fit (TTF) played a significant moderating role in the relationship between the use of generative artificial intelligence (GAI) and perceived English learning achievement. The results indicate that the association between GAI use and perceived learning outcomes is conditional on Task–Technology Fit: when technological functions are perceived to match task demands, the positive association between use behavior and perceived learning outcomes is significantly strengthened. This finding supports the core argument of Task–Technology Fit theory and extends it to the field of GAI-supported language learning, consistent with Walker et al. (2023) and Moghavvemi and Jam (2025). Importantly, high levels of self-reported GAI use are not automatically linked to perceived learning gains unless the technology is perceived to align well with specific language tasks. These findings emphasize that the perceived educational value of GAI depends not only on acceptance-related factors and sustained engagement with the technology, but also on the perceived alignment between AI functions and learning goals.

However, the TTF items in this study capture students’ general perceived fit between GAI and their English learning, rather than a task-type-specific and function-specific alignment. The moderating effect observed here should therefore be interpreted as evidence of overall perceived fit strengthening the use-achievement association, not as evidence of verified matches between differentiated GAI functionalities and differentiated language-learning tasks.

### 5.5. Comparison with Related Studies

The findings of this study both converge with and extend recent research on GAI adoption in language learning. Consistent with Yakubu et al. (2025) and Lai et al. (2024), performance expectancy, effort expectancy, and facilitating conditions emerged as significant predictors of GAI use behavior, reinforcing the applicability of the UTAUT framework in AI-enhanced educational contexts. The strong predictive role of AI self-efficacy aligns with Xu and Xiong (2026), who reported that AI self-efficacy mediated the relationship between AI literacy and behavioral intention among Chinese foreign language learners, further confirming that perceived capability is a robust factor associated with engagement with AI tools in language learning contexts.

Where this study departs from much of the existing literature is in its treatment of use behavior and Task–Technology Fit. Most UTAUT-based studies in AI-assisted education treat behavioral intention as the primary outcome, whereas the present study positions use behavior as a mediating mechanism linking acceptance factors to learning achievement (X. Hu & Gong, 2025; Xu & Xiong, 2026). Although recent studies have examined TTF in GAI adoption (Deng et al., 2025; Saifi et al., 2025), few have investigated its moderating role on the relationship between use behavior and learning achievement in language learning. The finding that the positive association between use behavior and perceived English learning achievement is strengthened under high Task–Technology Fit adds nuance to studies that report a direct, unconditional link between GAI use and positive outcomes, and supports the proposition that GAI is conditionally effective depending on the alignment between tool capabilities and task requirements (Yang & Sun, 2025).

### 5.6. Theoretical Implications

By integrating the Unified Theory of Acceptance and Use of Technology (UTAUT) with the Task–Technology Fit (TTF) theory in the context of generative artificial intelligence (GAI), this study extends the theoretical boundaries of technology adoption research in AI-supported learning. GAI is highly open and task-dependent, and its learning effects vary across different task contexts. By introducing TTF as a moderating variable, this study emphasizes that the perceived match between technological functions and learning task demands is an important condition under which GAI use is more strongly associated with students’ perceived learning gains, providing a more refined theoretical perspective for explaining when GAI is perceived as effective in supporting student learning.

This study positions use behavior, which reflects both current use and experience-based behavioral persistence (Ashraf et al., 2025; Lavidas et al., 2024), as the core mediating mechanism, contributing to a gradual theoretical shift in technology adoption research from purely intention-based explanations toward operationalizations that incorporate behavioral persistence and engagement. We acknowledge that this represents a partial rather than a complete departure from intention-based frameworks (X. Hu & Gong, 2025; Xue et al., 2024), given that our measurement of use behavior includes a continuance-orientation component, fully observed behavioral measures would be needed to complete this theoretical transition in future research. The results indicate that variables such as performance expectancy are not directly associated with perceived English learning achievement, but instead show indirect associations through students’ use behavior of GAI. This finding helps address a mechanistic gap in technology-enhanced learning research by clarifying how technology use is linked to students’ perceived English learning achievement through their reported learning behaviors, offering a more explanatory framework for understanding the pathway through which GAI may support language learning.

By incorporating artificial intelligence self-efficacy and perceived competitiveness, this study enriches and extends the theoretical meaning of UTAUT in higher-education GAI contexts. This extension helps embed technology adoption theory more closely into contemporary higher-education learning contexts and deepens the theoretical understanding of students’ self-reported strategic engagement with GAI tools.

### 5.7. Practical Implications

The following implications should be interpreted within the scope of the present study. The sample consisted of undergraduate students recruited through English-learning WeChat groups at five Chinese universities, who already had prior experience with GAI tools. The recommendations below are therefore most directly applicable to comparable contexts in which students are already familiar with and favorably disposed toward GAI, rather than to questions about initial adoption barriers among students with no prior GAI exposure, or to the broader higher-education population beyond the surveyed institutions.

Within the scope described above, the findings of this study offer several tentative practical considerations for educators and students working with GAI in higher-education language-learning contexts.

Teachers need to design instructional interventions around use behavior. The results suggest that enhancing students’ perceptions of the usefulness of AI or expressing general support for the technology may not be sufficient on its own; students’ engagement with GAI appears to play an important role in the association with perceived learning outcomes. Based on this result, English teachers may consider encouraging students to engage actively with GAI tools as part of their learning routines, while recognizing that the present findings reflect students’ self-reported use rather than externally observed behavior. For example, teachers can explicitly specify when and how AI should be used in the course, such as using it to optimize language expression during the writing process (Kasneci et al., 2023). Embedding AI use into specific learning tasks may help create conditions conducive to students’ ongoing engagement with GAI, which, based on the present findings, is positively associated with their perceived English learning achievement.

Teachers need to acknowledge competitive contexts and transform perceived competitiveness into constructive learning motivation. This study finds that perceived competitiveness is significantly associated with students’ use of GAI. This result provides important insights into the real-world motivations underlying students’ use of AI. Educators can deliberately guide this competitive motivation into constructive learning motivation (Bailey et al., 2024). Through peer discussions and reflective tasks, teachers can guide students to share how AI helps them improve learning strategies.

Teachers can design learning activities that align with Task–Technology Fit (R. Kim & Song, 2022). The results suggest that the positive association between use behavior and perceived English learning achievement appears to be stronger when AI functions are perceived to align well with specific learning tasks. Teachers should selectively introduce GAI according to different English learning goals. In courses that emphasize the development of writing ability, teachers can focus on using AI for multiple rounds of revision, feedback comparison, and language refinement. In contrast, when cultivating critical reading or oral communication skills, teachers need to carefully design the mode of use to avoid AI replacing core cognitive processes.

### 5.8. Limitations

This study has several limitations. The participants were drawn from five universities in China through convenience sampling via English-learning WeChat groups. Although the sample size was sufficient for structural equation modeling, the regional concentration and recruitment strategy may have introduced self-selection bias, as respondents may represent a subgroup with stronger learning motivation or greater engagement with technology (Bethlehem, 2010; Kline, 2023; Stone et al., 2024). The relatively small number of participants from natural science disciplines may also limit the examination of disciplinary differences in GAI application and related learning outcomes (Biglan, 1973; Y. Qu et al., 2024). All variables, including the outcome, were measured through self-report from a single source at a single time point, and self-reported learning outcomes may not accurately reflect actual performance (Deslauriers et al., 2019; H. Liu et al., 2025). This single-source, cross-sectional design means that, despite the use of Harman’s test and CFA-based discriminant validity evidence, some degree of common method variance cannot be entirely ruled out. The TTF items also captured a general sense of perceived fit rather than a task-type-specific and function-specific alignment, which limits the precision with which the moderating effect can be interpreted in terms of specific GAI functionalities or task types. Finally, this study focused solely on students’ perceptions without incorporating the perspectives of teachers or institutional stakeholders. Future research adopting a multi-stakeholder approach would provide a more comprehensive understanding of GAI integration in language learning (Selwyn, 2010; Zhang et al., 2025).

### 5.9. Future Research Directions

Future research can extend this study’s findings in several ways. Expanding the sample to include learners from different types of institutions and cultural backgrounds would help to examine the generalizability of the results and enable cross-cultural comparisons of generative artificial intelligence applications in language learning. Future studies could incorporate multiple stakeholders’ perspectives by including teachers’ instructional practices and relevant institutional and policy factors, thereby providing a more comprehensive understanding of how instructional design, teacher guidance, and organizational support are related to the mechanisms of AI-assisted language learning. Given that perceived English learning achievement in this study was measured through self-report items, future research should incorporate objective achievement indicators such as standardized test scores or course grades to provide a more robust assessment of learning outcomes. Similarly, use behavior could be supplemented with behavioral trace data such as system log records or usage frequency tracking to complement self-reported measures.

## 6. Conclusions

This study examined how undergraduate students’ use of generative artificial intelligence (GAI) is associated with perceived English learning achievement by integrating UTAUT and TTF theory. Eight hypotheses were tested. The results indicated that performance expectancy (H1), effort expectancy (H2), facilitating conditions (H3), perceived competitiveness (H4), and artificial intelligence self-efficacy (H5) were positively associated with students’ GAI use behavior. Use behavior played a mediating role in the relationships between each of the five antecedent variables and perceived English learning achievement (H6a-H6e), with mediation proportions ranging from 28.7% to 34.4%, suggesting partial rather than full mediation. A positive association between use behavior and perceived English learning achievement was also observed (H7). Task–Technology Fit moderated this association (H8), with a stronger link observed when GAI functions were more closely aligned with learning task requirements. The findings were consistent with the proposed theoretical model, though the cross-sectional design warrants caution in interpreting directional relationships.

## Figures and Tables

**Table 1:** Sample demographic characteristics.

Variables	Category	Frequencies	Percentage
Gender	Male	282	52.51%
	Female	255	47.49%
Grade	First	73	13.59%
	Second	166	30.91%
	Third	177	32.96%
	Fourth	121	22.53%
Field of Study	Natural Sciences	165	30.73%
	Social Sciences	372	69.27%
Total		537	100%

**Table 2:** Measurement items and sources.

Construct	Code	Measurement Item	Source
Performance Expectancy (PE)	PE1	I believe that using GAI helps me complete English learning tasks more quickly.	(Sha et al., 2025; Zheng et al., 2024)
	PE2	I believe that using GAI improves the quality of my English learning.	
	PE3	I believe that using GAI increases my efficiency in learning English.	
	PE4	If I use GAI tools, I will have a greater chance of achieving better results in my English courses.	
Effort Expectancy (EE)	EE1	I believe that I can easily learn how to use GAI for English learning.	(Sha et al., 2025; Zheng et al., 2024)
	EE2	I think GAI is easy to use.	
	EE3	Learning English using GAI is easy for me.	
	EE4	My interaction with GAI tools is clear and understandable.	
Facilitating Conditions (FC)	FC1	I have the resources necessary to use GAI for English learning.	(Ashraf et al., 2025; Mohd Rahim et al., 2022)
	FC2	I have the knowledge necessary to use GAI for English learning.	
	FC3	I have teachers who can support me when I encounter problems related to GAI.	
	FC4	My institution supports the use of GAI.	
	FC5	GAI is compatible with other technologies I use.	
Perceived Competitiveness (PC)	PC1	I believe that standing out in English courses requires more effort than others.	(Bailey et al., 2024; Canning et al., 2020)
	PC2	There is strong ranking pressure in my class.	
	PC3	I feel that everyone in English courses is striving to achieve higher grades than others.	
	PC4	I perceive strong competition among classmates.	
AI Self-Efficacy (AISE)	AISE1	I am confident in using GAI for learning.	(Hong, 2022; Y. Wang & Chuang, 2024)
	AISE2	I am willing to make efforts to learn knowledge and skills related to GAI.	
	AISE3	I am determined to promote my learning of GAI-related knowledge and skills.	
	AISE4	I try my best to learn knowledge and skills related to GAI.	
	AISE5	For me, learning GAI-related knowledge and skills is very important.	
Use Behavior (UB)	UB1	I am currently using GAI to support my English learning.	(Sha et al., 2025; Lavidas et al., 2024)
	UB2	I frequently use GAI to support my English learning.	
	UB3	I intend to continue using GAI for English learning.	
Perceived English Learning Achievement (ELA)	ELA1	GAI has helped improve my English learning.	(Ashraf et al., 2025; Dahri et al., 2024)
	ELA2	GAI has enhanced my understanding of English learning.	
	ELA3	GAI makes learning English easier for me.	
	ELA4	GAI has a positive impact on my overall English learning efficiency.	
Task–Technology Fit (TTF)	TTF1	GAI meets all my needs for learning English.	(Sha et al., 2025; Wu & Chen, 2017)
	TTF2	I find GAI very useful for developing my English skills.	
	TTF3	Using GAI fits well with my English learning practices.	

**Table 3:** Results of Harman’s Single-Factor Test.

Component	Initial Eigenvalues (Total)	% of Variance	Cumulative %	Rotation Sums of Squared Loadings (Total)
1	10.168	31.775	31.775	3.327
2	2.035	6.358	38.134	3.297
3	1.859	5.808	43.942	3.286
4	1.728	5.400	49.341	2.878
5	1.673	5.229	54.570	2.792
6	1.633	5.103	59.673	2.760
7	1.451	4.536	64.208	2.206

**Table 4:** Group differences across demographic variables.

Demographic Variables		Performance Expectancy (M ± SD)	Effort Expectancy (M ± SD)	Facilitating Conditions (M ± SD)	Perceived Competitiveness (M ± SD)	AI Self-Efficacy (M ± SD)	Use Behavior (M ± SD)	Task–Technology Fit (M ± SD)	Perceived English Learning Achievement (M ± SD)
Gender	Male	3.353 ± 0.787	3.384 ± 0.721	3.445 ± 0.683	3.408 ± 0.668	3.416 ± 0.727	3.597 ± 0.751	3.435 ± 0.759	3.296 ± 0.706
	Female	3.378 ± 0.753	3.397 ± 0.763	3.464 ± 0.691	3.430 ± 0.723	3.443 ± 0.763	3.728 ± 0.760	3.457 ± 0.728	3.365 ± 0.685
T		−0.371	−0.204	−0.324	−0.370	−0.425	−2.010 *	−0.337	−1.146
Grade	First	3.184 ± 0.868	3.103 ± 0.807	3.173 ± 0.803	3.255 ± 0.772	3.183 ± 0.809	3.330 ± 0.938	3.379 ± 0.719	3.190 ± 0.736
	Second	3.300 ± 0.736	3.417 ± 0.682	3.465 ± 0.643	3.398 ± 0.672	3.415 ± 0.756	3.679 ± 0.673	3.371 ± 0.763	3.319 ± 0.752
	Third	3.485 ± 0.727	3.484 ± 0.735	3.537 ± 0.626	3.484 ± 0.709	3.485 ± 0.687	3.727 ± 0.742	3.524 ± 0.743	3.390 ± 0.620
	Fourth	3.426 ± 0.766	3.451 ± 0.720	3.550 ± 0.673	3.484 ± 0.613	3.570 ± 0.709	3.807 ± 0.645	3.488 ± 0.731	3.368 ± 0.682
F		3.807 **	6.182 ***	7.132 ***	2.667 *	5.264 ***	8.214 ***	1.554	1.851
Field of Study	Natural Sciences	3.355 ± 0.777	3.395 ± 0.707	3.433 ± 0.684	3.402 ± 0.710	3.396 ± 0.749	3.615 ± 0.736	3.437 ± 0.763	3.297 ± 0.712
	Social Sciences	3.388 ± 0.801	3.377 ± 0.813	3.501 ± 0.691	3.455 ± 0.658	3.503 ± 0.728	3.760 ± 0.797	3.465 ± 0.698	3.402 ± 0.656
T		−0.458	−0.258	−1.044	−0.811	−1.545	−2.051 *	−0.393	−1.606

Note: M = mean; SD = standard deviation; T = independent-samples t value; F = one-way ANOVA F value. * *p* < 0.05; ** *p* < 0.01; *** *p* < 0.001. Effect sizes for gender and field of study comparisons (Cohen’s d) ranged from 0.018 to 0.177, and effect sizes for grade comparisons (η^2^) ranged from 0.009 to 0.044, all indicating small practical effects.

**Table 5:** Descriptive statistics and reliability assessment.

Variable	N	Mean	SE	Cronbach’s α	AVE	CR
Performance Expectancy (PE)	537	3.365	0.033	0.846	0.580	0.847
Effort Expectancy (EE)	537	3.390	0.032	0.839	0.565	0.839
Facilitating Conditions (FC)	537	3.454	0.030	0.868	0.570	0.870
Perceived Competitiveness (PC)	537	3.418	0.030	0.833	0.557	0.834
AI Self-Efficacy (AISE)	537	3.429	0.032	0.869	0.571	0.870
Use Behavior (UB)	537	3.659	0.033	0.833	0.611	0.834
Task–Technology Fit (TTF)	537	3.446	0.032	0.794	0.572	0.799
Perceived English Learning Achievement (ELA)	537	3.329	0.030	0.829	0.550	0.830

Note: N = sample size; SE = standard error; Cronbach’s α = Cronbach’s alpha coefficient; AVE = average variance extracted.

**Table 6:** Discriminant validity (Fornell–Larcker criterion).

	PE	EE	FC	PC	AISE	UB	ELA	TTF
PE	0.762							
EE	0.383	0.753						
FC	0.378	0.436	0.756					
PC	0.320	0.374	0.390	0.747				
AISE	0.357	0.395	0.450	0.430	0.756			
UB	0.368	0.399	0.393	0.374	0.417	0.791		
ELA	0.399	0.385	0.428	0.379	0.432	0.433	0.742	
TTF	0.268	0.330	0.366	0.339	0.386	0.353	0.375	0.756

Note: PE—Performance Expectancy; EE—Effort Expectancy; FC—Facilitating Conditions; PC—Perceived Competitiveness; AISE—AI Self-Efficacy; UB—Use Behavior; ELA—Perceived English Learning Achievement; TTF—Task–Technology Fit.

**Table 7:** Model fit indices.

CMIN	DF	CMIN/DF	GFI	IFI	TLI	CFI	RMSEA
626.733	361	1.736	0.922	0.964	0.959	0.964	0.037

Note: CMIN = minimum discrepancy (chi-square) statistic; DF = degrees of freedom; CMIN/DF = normed chi-square; GFI = goodness-of-fit index; IFI = incremental fit index; TLI = Tucker–Lewis index; CFI = comparative fit index; RMSEA = root mean square error of approximation.

**Table 8:** Main and moderating effects.

Variables		Use Behavior			Perceived English Learning Achievement		
		1	2	3	4	5	6
Control variables	Gender	0.096 *	0.079 *	0.078 *	0.052	0.019	0.024
	Grade	0.193 ***	0.088 *	0.091 *	0.091 *	−0.040	−0.027
	Field of Study	0.084 *	0.062	0.063 **	0.067	0.029	0.024
Independent variables	PE		0.144 ***	0.137 ***		0.155 ***	0.154 ***
	EE		0.161 ***	0.146 ***		0.093 *	0.076
	FC		0.121 **	0.100 *		0.154 ***	0.128 **
	PC		0.135 ***	0.118 **		0.104 **	0.089 *
	AISE		0.170 ***	0.145 ***		0.156 ***	0.138 **
Mediating variable	UB					0.178 ***	0.166 ***
Moderating variable	TTF			0.127 **			0.132 ***
	UB × TTF						0.114 ***
R^2^		0.051	0.314	0.327	0.015	0.347	0.370
△R^2^			0.263	0.012		0.332	0.013

Note: Standardized regression coefficients (β) are reported. PE = performance expectancy; EE = effort expectancy; FC = facilitating conditions; PC = perceived competitiveness; AISE = artificial intelligence self-efficacy; UB = use behavior; TTF = Task–Technology Fit; UB × TTF = interaction effect between use behavior and task–technology fit; R^2^ = explained variance; ΔR^2^ = incremental variance explained. * *p* < 0.05; ** *p* < 0.01; *** *p* < 0.001.

**Table 9:** Bootstrap results of mediation effects.

Effect Type	Path	Effect Estimate	Relative Mediation Effect (%)	Bias-Corrected 95%CI		*p*
				Lower	Upper	
Indirect effect	Performance Expectancy → Use Behavior → Perceived English Learning Achievement	0.110	30.5%	0.076	0.148	<0.001 (CI does not include 0)
Indirect effect	Effort Expectancy → Use Behavior → Perceived English Learning Achievement	0.125	34.4%	0.090	0.164	<0.001 (CI does not include 0)
Indirect effect	Facilitating Conditions → Use Behavior → Perceived English Learning Achievement	0.125	28.7%	0.088	0.164	<0.001 (CI does not include 0)
Indirect effect	Perceived Competitiveness → Use Behavior → Perceived English Learning Achievement	0.127	33.4%	0.097	0.168	<0.001 (CI does not include 0)
Indirect effect	AI Self-Efficacy → Use Behavior → Perceived English Learning Achievement	0.120	29.6%	0.084	0.159	<0.001 (CI does not include 0)

Note: CI = confidence interval; Lower and Upper refer to the lower and upper bounds of the bias-corrected 95% confidence interval obtained through bootstrap resampling. An indirect effect is statistically significant when the confidence interval does not include zero.

## Data Availability

The original contributions presented in this study are included in the article. Further inquiries can be directed to the corresponding author.
